# On the Role of the Striatum in Response Inhibition

**DOI:** 10.1371/journal.pone.0013848

**Published:** 2010-11-04

**Authors:** Bram B. Zandbelt, Matthijs Vink

**Affiliations:** Rudolf Magnus Institute of Neuroscience, Department of Psychiatry, University Medical Center Utrecht, Utrecht, The Netherlands; University of Barcelona, Spain

## Abstract

**Background:**

Stopping a manual response requires suppression of the primary motor cortex (M1) and has been linked to activation of the striatum. Here, we test three hypotheses regarding the role of the striatum in stopping: striatum activation during successful stopping may reflect suppression of M1, anticipation of a stop-signal occurring, or a slower response build-up.

**Methodology/Principal Findings:**

Twenty-four healthy volunteers underwent functional magnetic resonance imaging (fMRI) while performing a stop-signal paradigm, in which anticipation of stopping was manipulated using a visual cue indicating stop-signal probability, with their right hand. We observed activation of the striatum and deactivation of left M1 during successful versus unsuccessful stopping. In addition, striatum activation was proportional to the degree of left M1 deactivation during successful stopping, implicating the striatum in response suppression. Furthermore, striatum activation increased as a function of stop-signal probability and was to linked to activation in the supplementary motor complex (SMC) and right inferior frontal cortex (rIFC) during successful stopping, suggesting a role in anticipation of stopping. Finally, trial-to-trial variations in response time did not affect striatum activation.

**Conclusions/Significance:**

The results identify the striatum as a critical node in the neural network associated with stopping motor responses. As striatum activation was related to both suppression of M1 and anticipation of a stop-signal occurring, these findings suggest that the striatum is involved in proactive inhibitory control over M1, most likely in interaction with SMC and rIFC.

## Introduction

The ability to stop a response is crucial in everyday life. The stop-signal paradigm [1] provides a framework for investigating the processes underlying stopping. In this paradigm, go-signals requiring a response are infrequently followed by a stop-signal, indicating that the planned response should be stopped. Stopping performance depends on the outcome of an interactive race between a Go process (activated by the go-signal) building up to response threshold and a Stop process (activated by the stop-signal) that can inhibit the Go process [2]. The neural correlates of these Go and Stop processes have been found in the higher motor centers for eye movements [3], [4], and such Go and Stop units are thought to be present in the primary motor cortex (M1) as well [5].

Converging lines of evidence suggest that a fronto-basal ganglia network is involved in controlling such Go and Stop units [for review, see [6]]. The striatum, the main input station of the basal ganglia, is considered an important region for stopping. Specifically, functional neuroimaging studies observe increased striatum activation during successful versus unsuccessful stopping [7], [8], [9], [10], [11], when comparing short to long stop-signal reaction times [12], and with a parametric increase in stop-signal probability [7], [13]. Meta-analyses of functional neuroimaging studies of response inhibition confirm that the striatum is commonly recruited during stopping [14], [15], [16]. Clinical populations characterized by striatum dysfunction have stopping impairments [13], [17], [18], [19], [20]. Finally, striatum lesions cause stopping impairments in rats [21].

Three hypotheses have been put forward regarding the meaning of stopping-related activation of the striatum. First, it may reflect suppression of response-related M1 activation, as striatum activation and M1 deactivation co-occur with successful stopping [7], [8]. Second, it may indicate anticipation of a stop-signal occurring, given that striatum activation and response delaying in order to improve stopping performance co-occur with increasing stop-signal probability [7]. Third, it may reflect a slower build-up of the Go process to response threshold, which would allow the Stop process sufficient time to cancel the response [8]. We refer to these concepts as the response suppression, stop-signal anticipation, and response build-up hypotheses, respectively.

**Figure 1:**
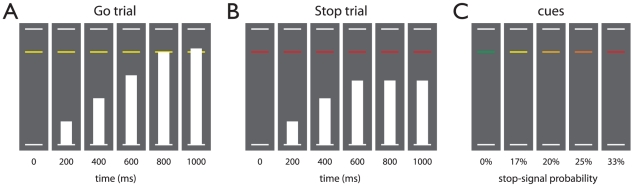
Schematic of the stop-signal anticipation task. Three horizontal lines formed the background displayed continuously during the task. (A) In each trial, a bar moved at constant speed from the bottom up, reaching the middle line in 800 ms. The main task was to stop the bar as close to the middle line as possible by pressing a button with the right thumb. These trials are referred to as Go trials. (B) In a minority of trials, the bar stopped moving automatically before reaching the middle line, indicating that a response had to be stopped. These trials are referred to as Stop trials. Stop-signal onset was adjusted in steps of 25 ms based on stopping performance, according to a 1-up-1-down staircase procedure (see Methods section). (C) The probability that a stop-signal would occur was manipulated across trials and was indicated by the color of the target response line. There were five stop-signal probability levels: 0% (green), 17% (yellow), 20% (amber), 25% (orange), and 33% (red).

Here, we investigate the role of the striatum in stopping, testing the hypotheses outlined above with a novel stop-signal paradigm (Fig. 1), in which stop-signal probability was manipulated using a visual cue. This enabled the measurement of response strategy adjustments in anticipation of stop-signals. Furthermore, to constrain waiting strategies that may limit the validity of the stop-signal paradigm [22], subjects were required to make timed rather than speeded responses [23]. We tested the hypotheses outlined above, using fMRI subtraction and psychophysiological interaction (PPI) analyses (Table 1). Specifically, we predict that if the striatum suppresses M1, striatum activation levels during successful stopping may be proportional to the amount of M1 deactivation. If the striatum is involved in stop-signal anticipation, then activation should increase as a function of stop-signal probability. It may very well be that the striatum signals the current context (i.e. stop-signal probability) to the cortex to guide behavior, for example, to enhance stop-signal monitoring by the right inferior frontal cortex (rIFC) and right temporoparietal junction (rTPJ) [24] or to delay responding via the rIFC or the supplementary motor complex (SMC), as stimulation of these areas improves stopping performance by delaying responses [25], [26]. We therefore predict that if the striatum is involved in stop-signal anticipation, striatum activation during successful stopping may be associated with activation in SMC, rIFC, and rTPJ. Finally, if striatum activation reflects response build-up speed, it should be proportional to response time on Go trials.

**Table 1:** Hypotheses.

	Stop-related activation of the striatum	Go-related activation of the striatum	Functional connectivity
Response suppression hypothesis	StopSuccess > StopFailure	-	Negative PPI of striatum with M1 for StopSuccess > StopFailure
Stop-signal anticipation hypothesis	StopSuccess > StopFailure	Parametric effect of stop-signal probability	Positive PPI of striatum with SMC, rIFC and rTPJ for StopSuccess > StopFailure
Response build-up hypothesis	StopSuccess > StopFailure	Parametric effect of response time	-

M1, primary motor cortex; PPI, psychophysiological interaction; rIFC, right inferior frontal gyrus; SMC, supplementary motor complex;

## Methods

### Ethics statement

This study was approved by the University Medical Center Utrecht ethics committee. All participants gave written informed consent according to procedures approved by this committee.

### Participants

24 healthy volunteers (mean age 22.2 years, range 19–26; 18 females) participated in this study. All participants were right-handed, had normal or corrected-to-normal vision, and did report no history of neurological or psychiatric illness.

### Stop-signal anticipation task

Participants peformed the stop-signal anticipation task (Fig. 1), a paradigm based on the stop-signal task [1], [7] and Slater-Hammel task [23], [27]. Three horizontal lines displayed one above the other, the middle line located 4/5 of the distance from the lower to the upper line, formed the background that was displayed continuously throughout the task. On each trial, a bar moved at a constant speed from the lower line towards the upper line, reaching the middle line in 800 ms. The main task was to stop the bar as close to the middle line as possible, by pressing a button with the right thumb (i.e. Go trial). Thus, the target response time was 800 ms. Stop trials were identical to Go trials, except that the bar stopped moving automatically before reaching the middle line, indicating that a response had to be suppressed (i.e. stop-signal). The probability that such a stop-signal would appear was manipulated across trials and could be anticipated on the basis of the color of the middle line (green, 0%; yellow, 17%; amber, 20%; orange, 25%; red, 33%).

The stop-signal onset time was initially set to 550 ms (i.e. 250 ms before the target response time) for all stop-signal probability levels. During the experiment, stop-signal onset time was adjusted (in steps of 25 ms) depending on stopping performance and for each stop-signal probability level separately. Specifically, if stopping was successful on the previous Stop trial, then stopping was made more difficult by shifting the stop-signal onset time 25 ms towards the target response time. The process was reversed when stopping failed. This ensures roughly equal numbers of successful and unsuccessful Stop trials.

Trials were presented in baseline and experimental blocks consisting of 12 to 15 trials, with an intertrial interval of 1000 ms. Baseline blocks consisted of Go trials with stop-signal probability of 0%, indicated to the subject by green stop-signal probability cues. Experimental blocks contained Go trials with stop-signal probability >0% (non-green cues) and Stop trials (non-green cues). Specifically, Stop trials were pseudorandomly interspersed between Go trials and stop-signal probability was manipulated across trials. We ran simulations prior to the experiment to determine the optimal trial order, such that correlations between the different model regressors was sufficiently low to allow for reliable estimation of parameter estimates. In total, 234 Go trials with stop-signal probability of 0%, 180 Go trials with stop-signal probability >0% (yellow, n = 30; amber, n = 48; orange, n = 54; red, n = 48), and 60 Stop trials (yellow, n = 6; amber, n = 12; orange, n = 18; red, n = 24) were presented. Two rest blocks of 24 s each, displaying the background only, were implemented at one-third and two-thirds of the task, respectively. The total task duration was 16 m 36 s.

Participants were trained on the stop-signal anticipation task before the fMRI experiment. We instructed participants that the Go task and Stop task were equally important and that it would not always be possible to suppress a response when a stop-signal occurred. We informed participants that stop-signals would never appear on trials with a green cue and that stop-signals could occur on trials with non-green cues. Participants were told that stop-signals were least likely in the context of a yellow cue and most likely in the context of a red cue, with the amber and orange cues coding intermediate stop-signal probabilities.

### Image acquisition

The experiment was performed on a 3.0 T Philips Achieva MRI scanner (Philips Medical Systems, Best, the Netherlands) at the University Medical Center Utrecht. Head motion was restricted using a vacuum cushion and foam wedges. Images were acquired using an eight-channel sensitivity-encoding (SENSE) parallel-imaging head coil. Whole-brain T2*-weighted echo planar images (EPI) with blood-oxygen level-dependent (BOLD) contrast (622 volumes; 30 slices per volume; interleaved acquisition; repetition time, 1600 ms; echo time, 23.5 ms; field of view: 256×208 mm; flip angle  = 72.5°; 64×51 matrix; 4×4 mm in-plane resolution; 4 mm slice thickness; SENSE-factor, 2.4 (anterior-posterior)) oriented in a transverse plane tilted 20° over the left-right axis were acquired in a single run. The first six images were discarded to allow for T1 equilibration effects. A whole-brain three-dimensional fast field echo T1-weighted scan (150 slices; repetition time  = 8.4 ms; echo time  = 3.8 ms; flip angle  = 8°; field of view, 288×252×185 mm; voxel size: 1 mm isotropic) was acquired for within-subject registration purposes.

### Data analysis

Behavioral data were analyzed using custom written software in Matlab 7 (Mathworks Inc., Natick, MA, USA). Response times (for Go) and accuracy were calculated for each stop-signal probability level separately. The stop-signal reaction time (SSRT) was calculated across all stop-signal probability conditions using the integration method [28]. We also computed inhibition functions for each subject, depicting the proportion of Stop trials in which stopping succeeded for each stop-signal onset time (collapsed across stop-signal probability levels). Go trials with response times of more than 1.5 times the interquartile range away from the 25^th^ and 75^th^ percentiles of the response time distribution of each stop-signal probability level were defined as outliers.

Image data were preprocessed and analyzed using Statistical Parametric Mapping 5 (SPM5) software (http://www.fil.ion.ucl.ac.uk/spm/software/spm5/) running in Matlab 7 (Mathworks Inc., Natick, MA, USA). Images were converted from PAR/REC to NifTI-1 format. Functional images were corrected for differences in acquisition times across slices, resampling all slices in time relative to the fifteenth slice using Fourier interpolation. To adjust for head motion, functional images were registered to the mean image using 4^th^-degree B-spline interpolation [29]. Estimated motion parameters were inspected to ensure that absolute motion over the course of the experiment did not exceed 4 mm and that the maximum image-to-image motion was never more than 1 mm. The anatomical image was co-registered to the mean functional image using the mutual information criteria method and segmented and normalized to the International Consortium for Brain Mapping template using linear and non-linear deformations [30], [31]. The normalization parameters were applied to the functional and anatomical images. Functional images were spatially smoothed using an 6-mm full-width at half-maximum Gaussian kernel. The T1-weighted images were skull-stripped using an automated brain extraction method [32].

Statistical analysis was performed within the framework of the general linear model and followed a two-level procedure. First-level statistical analysis involved modeling of StopSuccess, StopFailure, and Go trials with stop-signal probability >0% (conditions of interest), as well as rest and outlier trials (conditions of no interest) for each subject. We also included two parametric regressors modeling response time and stop-signal probability level of Go trials. Post-hoc analyses revealed that the correlation between the parametric regressors was sufficiently low to enable reliable estimation of parameter estimates. Go trials with stop-signal probability of 0% were not explicitly modeled and therefore constituted an implicit baseline. Regressors were created by convolving delta functions coding for response time (or target response time for StopSuccess trials) with a canonical hemodynamic response function. We accounted for residual head motion effects by including the motion parameters from the realignment procedure into the statistical model. Time series statistical analysis was performed using restricted maximum likelihood. Low frequency drifts were controlled using a discrete cosine transform with cutoff of 128 s. Serial correlations in the fMRI signal were estimated using restricted maximum likelihood estimates of variance components using a first-order autoregressive model. The resulting non-sphericity was used to form maximum-likelihood estimates of the activations. Contrast images were generated for the comparisons (1) StopSuccess versus StopFailure, (2) StopSuccess verusus Go, (3) parametric effect of stop-signal probability on Go, and (4) parametric effect of response time on Go.

First-level contrast images were analyzed in a second-level random-effects analysis, using one-sample *t*-tests. Group statistical parametric maps were tested for significance using cluster-level inference (cluster-defining threshold, P<0.001; cluster probability of P<0.05, family wise error-corrected for multiple comparisons). Reported local maxima correspond to Montreal Neurological Institute space. Activations were localized according to anatomical landmarks identified from the mean T1-weighted structural image of all participants with the aid of a human brain atlas [33] and a probabilistic atlas of human brain structures [34].

In an additional statistical analysis, we classified Go trials into eight different regressors according to stop-signal probability (17%, 20%, 25%, 33%) and response time bin (slow and fast). This analysis was conducted to confirm the results from the parametric analysis of stop-signal probability and response time, as parametric modulators may have reduced statistical power [35]. First-level model construction and estimation was performed as described above. Contrast images were generated for each combination of stop-signal probability level and response time bin. They were entered into a second-level random effects full factorial analysis, to test the main effects of stop-signal probability and response time bin, as well as the interaction between these factors. In addition, we performed a region-of-interest analysis by extracting mean parameter estimates from four striatal regions (see Results) for all the contrast images. For each ROI, we performed a repeated-measures analysis of variance with stop-signal probability and reaction time bin as factors.

We investigated effective connectivity of the striatum in a psychophysiological interaction (PPI) analysis [36], [37], testing for condition-specific (StopSuccess versus StopFailure) changes in coupling between the striatum and the rest of the brain that occurred over and above any main effects of context and striatal functional connectivity. A significant PPI entails a change in the slope of the regression of activity in a ‘sink’ region (e.g. left M1) onto activity in a ‘seed’ region (e.g. left striatum) from one condition (e.g. StopSuccess) to another (e.g. StopFailure). In the present analysis, a positive PPI indicates that the slope of the regression line is more positive in the StopSuccess condition as compared to the StopFailure condition, whereas a negative PPI indicates that this slope is more negative in the StopSuccess condition relative to the StopFailure condition. We performed PPI analyses using seed regions in the striatum, based on the local maxima from the one-sample *t*-test testing the contrast StopSuccess versus StopFailure (see Results section). For each subject and seed region, we extracted the first eigenvariate of the fMRI signal (adjusted for head motion) from a sphere with 8-mm radius centered around the local maximum. We obtained estimates of neural activity in this region by hemodynamic deconvolution using parametric empirical Bayes (physiological vector). The psychological vector was a delta function coding for onset times of StopSuccess (1) and StopFailure (-1) trials. We computed the PPI by taking the product of the physiological and psychological vectors at each point in time. The physiological, psychological and PPI vectors were then convolved with the canonical hemodynamic response function and entered as regressors in a first-level general linear model. Similar to the standard first-level general linear model, correlations between the three regressors were low, enabling reliable estimation of parameter estimates. Time series statistical analysis was identical to that described above. A contrast image was created for the PPI. The contrast images of all participants were tested at the second level in a one-sample t-test to identify regions showing a positive PPI or negative PPI, again using cluster-level inference.

## Results

### Behavior

**Figure 2:**
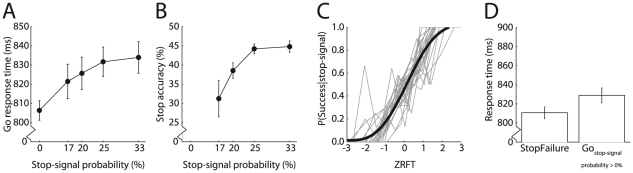
Stop-signal anticipation task performance. Stop-signal probability effects on (A) Go trial response time and (B) Stop trial accuracy. (C) Individual (grey) and group mean (black) normalized inhibition functions. The normalized inhibition function plots the proportion of successfully stopped responses as a function of the relative finishing times (RFT) of the stop and response processes, expressed as a Z-score (ZRFT). ZRFT was calculated as described in [1], collapsed across stop-signal probability levels. Positive ZRFT values represent early stop-signal onset, negative ZRFT values indicate late stop-signal onset. A cumulative Weibull function was fit to the group mean standardized inhibition function. (D) Mean response time on StopFailure trials and Go trials with stop-signal probability >0%. Error bars indicate 95% confidence intervals.

Response times on baseline Go trials (stop-signal probability of 0%) were close to the target response time of 800 ms (806 ms, 801–812 ms; mean, 95% C.I.), indicating that participants were able to perform the response task accurately. Response times on Go trials in which stop-signals could occur (829 ms, 821–837 ms; mean, 95% C.I.) were significantly higher than response times on baseline Go trials (paired t-test, t(23) = 9.13, P<0.001). Moreover, response times increased linearly as a function of stop-signal probability (Fig. 2A; linear contrast, F(1,23) = 29.07, P<0.001), suggesting that participants slowed responding according to the degree to which they anticipated stop-signals. This interpretation was confirmed by the finding that accuracy on Stop trials also increased linearly as a function of stop-signal probability (Fig. 2B; linear contrast, F(1,23) = 44.86, P<0.001).

The SSRT was longer than usually reported (326 ms, 320–333 ms; mean, 95% C.I.). Nevertheless, the data were in agreement with assumptions of the race model [1]. First, stop rate decreased with later stop-signal onset (Fig. 2C). Second, response times on StopFailure trials were faster than on Go trials in which stop-signals could occur (Fig. 2D; paired t-test, t(23) = 7.43, P<0.001).

### Functional MRI

#### Successful stopping versus failing to stop

**Figure 3:**
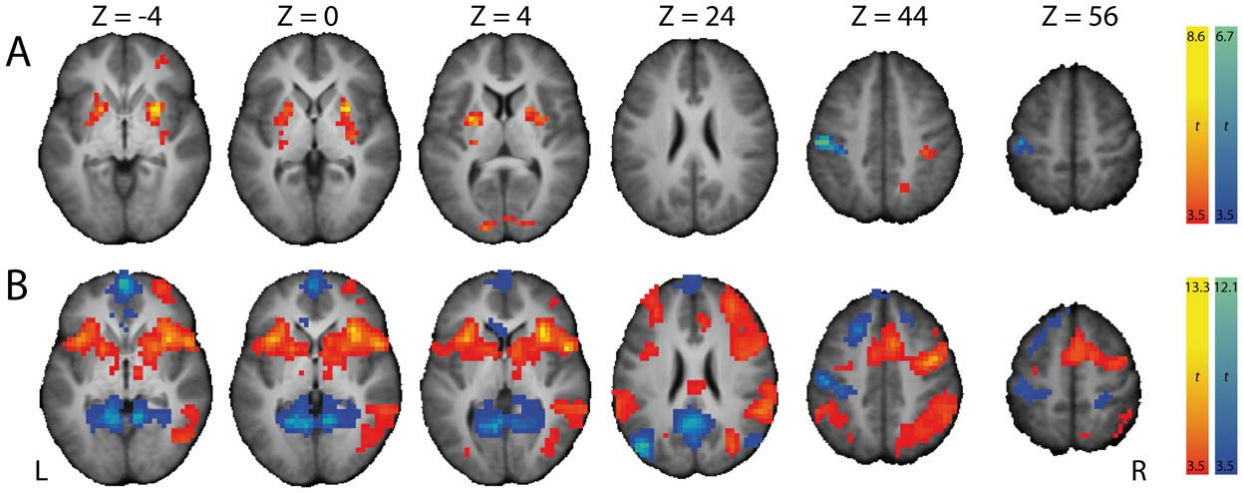
Brain regions with significant BOLD signal changes when contrasting (A) StopSuccess with StopFailure and (B) StopSuccess with Go trials with stop-signal probability of 0%. Warm colors represent activation during StopSuccess trials, cool colors represent deactivation during StopSuccess trials. Significant clusters of activation (P<.05, FWE-corrected) are displayed on the normalized and skull-stripped group-average brain (neurological orientation). L, left; R, right.

We first identified brain regions showing stopping-related activation by contrasting fMRI signals from StopSuccess and StopFailure trials (Table 1). Successful stopping significantly activated clusters in the left and right striatum (Fig. 3A) and both clusters were restricted to the putamen (Table S1). The left and right striatum were also activated when contrasting StopSuccess trials and Go trials with stop-signal probability of 0% (Fig. 3B). Importantly, these findings support the notion that the striatum is involved in stopping a planned response. Other regions that were activated included occipital areas, the right supramarginal gyrus, and the right orbitofrontal cortex (StopSuccess > StopFailure), as well as the supplementary motor complex, right inferior frontal cortex, and bilateral temporoparietal junction (StopSuccess > Go stop-signal probability of 0%). The left sensorimotor cortex, including M1, was deactived during successful stopping, consistent with suppression of a right-hand response.

To further examine the role of the striatum in stopping, we analyzed condition-specific changes (StopSuccess > StopFailure and StopSuccess < StopFailure) in cortico-striatal effective connectivity and the influence of stop-signal anticipation and response speed on striatum activation during Go trials (Table 1). Stopping outcome-dependent changes in cortico-striatal effective connectivity were investigated in four psychophysiological interaction (PPI) analyses. The striatal seeds for these PPI analyses were based on the previous analysis testing for stopping-related activation (Table S1, StopSuccess > StopFailure). Specifically, we selected the two most significant local maxima in the left striatum (-20 8 -4, left ventral putamen; -28 0 8, left dorsal putamen) and right striatum (28 8 -4, right ventral putamen; 20 4 12, right dorsal putamen).

#### Response suppression hypothesis

**Figure 4:**
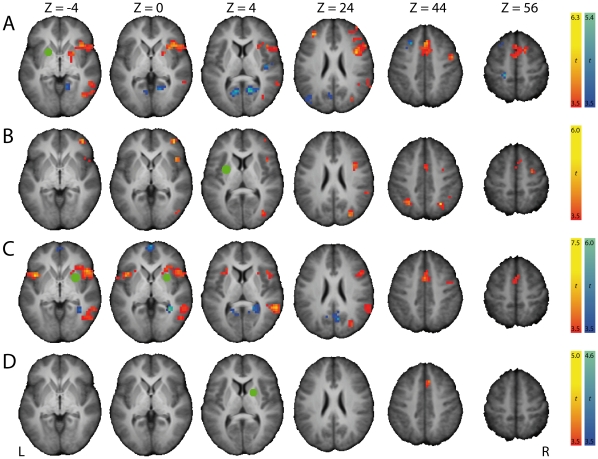
Brain regions with significant differences in coupling with the striatum as a function of Stop trial outcome (StopSuccess vs StopFailure). The statistical parametric maps shown are the result from four psychophysiological interaction (PPI) analyses, each for a different seed region in the striatum (shown as a green dot), defined as 8-mm spheres around the two most significant local maxima of the left and right striatum clusters of the StopSuccess vs StopFailure contrast, being (A) -20 8 -4, (B) -28 0 8, (C) 28 8 -4, and (D) 20 4 12. Warm colors indicate a positive PPI, cool colors indicate a negative PPI. Significant clusters of activation (P<.05, FWE-corrected) are displayed on the normalized and skull-stripped group-average brain (neurological orientation). L, left; R, right.

**Figure 5:**
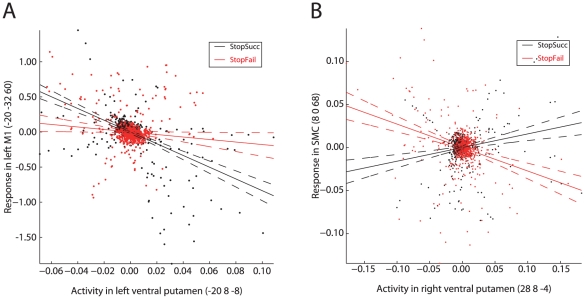
Example psychophysiological interaction (PPI) plots. (A) Example of a negative PPI between left ventral putamen and left primary motor cortex (M1) in one of the participants. (B) Example of a positive PPI between right ventral putamen and supplementary motor complex (SMC) in one of the participants. StopSucc, successful Stop trials. StopFail, unsuccessful Stop trials.

The co-occurrence of left M1 deactivation and bilateral activation of the striatum during successful versus unsuccessful stopping (Fig. 3A) does not necessarily mean that activation of the striatum is linked to deactivation of M1. We therefore performed a PPI analysis and found that activation of the left ventral putamen (-20 8 -4) during successful stopping was proportional to the level of deactivation in left M1 (Fig. 4A, Table S2). Figure 5A shows this negative PPI in a representative subject. This result indicates that stronger activation of the left ventral putamen during successful stopping was associated with stronger deactivation of left M1. The other PPI analyses, seeded in the left dorsal and right ventral and dorsal putamen, did not show significant negative PPIs with left M1 (Fig. 4B-D, Table S1). However, lowering the threshold (height, P<.01, uncorrected; extent, 5 voxels) revealed negative PPIs with left M1 for the left dorsal putamen and right ventral putamen. These data provide indirect support for a role for the striatum in suppressing left M1 during successful stopping.

#### Stop-signal anticipation hypothesis

As indicated by the behavioral results, participants improved stopping performance through response slowing (i.e. anticipation). If the striatum is implicated in stop-signal anticipation, then we expect its activation to increase with stop-signal probability. This response slowing may be induced by modulation of activity in SMC and rIFC, as stimulation of these areas improved stopping performance by delaying responses [25], [26]. If true, then striatum activation should show a stronger coupling with activation of SMC and rIFC during successful stopping (i.e. positive PPI), in which anticipation is at its maximum (Table 1).

**Figure 6:**
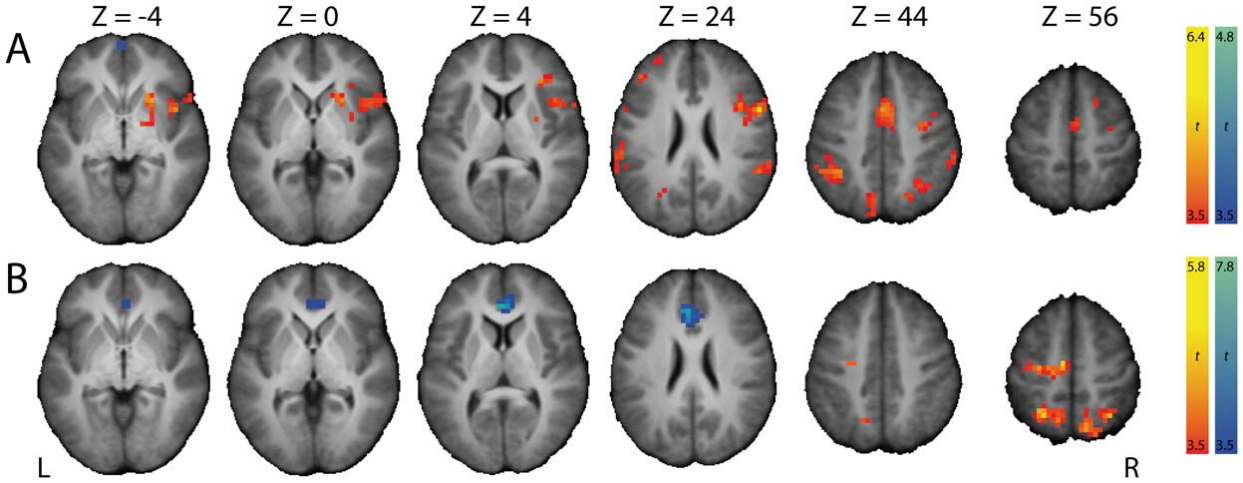
Brain regions with significant parametric effects of (A) stop-signal probability and (B) response time on the BOLD signal during Go trials. Significant clusters of activation (P<.05, FWE-corrected) are displayed on the normalized and skull-stripped group-average brain (neurological orientation). L, left; R, right.

Similar to the reaction time effect, we found a parametric effect of stop-signal probability on activation of a right anterior striatum cluster during Go trials, suggesting that right striatum activation reflects stop-signal anticipation (Fig. 6A, Table S3). We did not observe a significant effect of stop-signal probability in the left striatum at the cluster level, but we did at a more liberal threshold (height, P<.01, uncorrected; extent, 5 voxels). Interestingly, activation increased parametrically with stop-signal probability also in the rIFC and SMC, extending into the cingulate motor area and bilateral parietal regions. Given that the statistical model included a parametric regressor coding for Go response time (that had a low correlation with the stop-signal probability regressor, see Methods), these stop-signal anticipation-related activations were not confounded by trial-to-trial variations in response speed.

The PPI analyses showed that there was a significant positive coupling between striatum activation and activation of SMC, rIFC and a number of other cortical areas during successful stopping (Fig. 4A-D, Table S2). We found the most pronounced effects for the right ventral striatum seed (Fig. 4C). Remarkably, this seed (28 8 -4) was close to the local maximum of the right striatum cluster (24 16 -4), showing a parametric effect of stop-signal probability on Go trial activation (Fig. 6A, Table S3). Figure 5B shows the positive PPI between the right ventral putamen and the SMC in a representative subject. In an additional analysis, we tested for reverse PPIs with seeds in the SMC and rIFC. None of these PPI analyses showed a significant coupling with striatum activation during successful stopping. At a more liberal threshold (height, P<.01, uncorrected; extent, 5 voxels), however, there was a positive PPI between the SMC and left dorsal caudate and between the rIFC and right dorsal putamen. Together, these data indicate that striatum is involved in stop-signal anticipation.

#### Response build-up hypothesis

We already showed that the increased activation of the right striatum with stop-signal probability is not confounded by response time. However, there may be an effect of response time on left striatum activation. We therefore tested the response build-up hypothesis by assessing whether striatum activation on Go trials increases linearly with response time, while at the same time controlling for any effects of stop-signal anticipation (Table 1).

There were no significant effects of Go response time on striatum activation. In contrast, activation in left M1 and the left and right superior parietal lobule increased, whereas activation in anterior cingulate and right insula decreased as a function of response time (Fig. 6B, Table S3). Even at a lower threshold (height, P<.01, uncorrected; extent, 5 voxels) there were no significantly activated clusters in the striatum.

**Figure 7:**
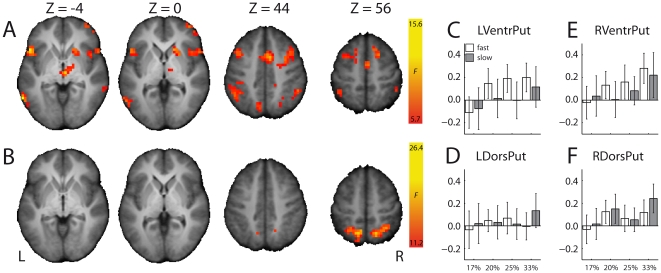
Results from the repeated-measures analysis of variance testing effects of stop-signal probability and response time bin on BOLD signal changes during Go trials. (A) Brain regions showing a significant main effect of stop-signal probability (17%, 20%, 25%, 33%) on BOLD signal changes during Go trials. (B) Brain regions showing a significant main effect of response time bin (fast, slow) on BOLD signal changes during Go trials. Significant clusters of activation (P<.05, FWE-corrected) are displayed on the normalized and skull-stripped group-average brain (neurological orientation). L, left; R, right. (C–F) Effects of stop-signal probability (horizontal axis) and response time bin (white, fast response times; grey, slow response times) on BOLD signal changes (vertical axis, arbitrary units) during Go trials in (C) left ventral putamen, (D) left dorsal putamen, (E) right ventral putamen, and (F) right dorsal putamen.

To address the possibility that this null finding reflects a lack of statistical power associated with parametric modulators [35], we performed an additional analysis. In this analysis, Go trials were classified according to stop-signal probability (17%, 20%, 25%, 33%) and response time bin (slow and fast). We investigated the main effects of stop-signal probability and response time bin and the interaction between the two in a whole-brain voxel-wise analysis and in a region-of-interest (ROI) analysis. The ROIs were the four spheres that were used as seeds in the PPI analyses. The whole-brain voxel-wise analyses revealed significant main effects of stop-signal probability (Fig. 7A) and response time (Fig. 7B). The network activated for each of these main effects was strikingly similar to the network activated in the parametric analyses testing for stop-signal probability and response time (Fig. 6). Again, there were no striatal clusters showing a significant effect of response time. Note that the left M1 cluster that reached significance in the parametric analysis, did not reach significance in the additional repeated-measures analysis (P = .065). There were also no clusters showing a stop-signal probability by response time interaction effect on activation. The ROI analyses (Fig. 7C-F) revealed that none of the striatum clusters showed a main or interaction effect of response time (all P>.12). However, there was a significant effect of stop-signal probability on activation in the left ventral putamen (F(2.9,67.2) = 3.52, P = .02) and the right ventral and dorsal putamen (F(2.6,59.6) = 3.60, P = .02 and F(2.3,53.5) = 3.37, P = .04). These findings replicate those from the parametric effect of stop-signal probability and extend them by showing also an effect in the left ventral striatum. Collectively, these findings indicate that striatum activation during successful stopping unlikely reflects a difference in response build-up speed between successful and unsuccessful stopping.

## Discussion

This study examined the role of the striatum in stopping using functional MRI. Based on previous findings, we hypothesized that striatum activation during successful stopping could reflect either suppression of the primary motor cortex (M1), anticipation of a stop-signal occurring, or a slower response build-up (Table 1). We used a stop-signal paradigm, in which stop-signal anticipation was manipulated using a visual cue indicating stop-signal probability. As expected, Go response time increased as a function of stop-signal probability (Fig. 2), confirming earlier findings [7], [13], [38], [39].

The present findings provide support for the response suppression hypothesis. We observed bilateral activation of the striatum and deactivation of left M1 during successful versus unsuccessful stopping (Fig. 3A), in line with previous reports [7], [8], [9], [10], [11]. Moreover, the degree of left M1 deactivation during successful stopping was proportional to activation of the left striatum (Fig. 4A). Note that the rIFC and SMC were not activated in this contrast, corroborating others [7], [8], [10], [40]. Perhaps, timing of activity rather than activation level dissociates successful from unsuccessful stopping in these regions.

The stop-signal anticipation hypothesis was also confirmed by our findings. Our finding of striatal activation during successful stopping (Fig. 3) is in accordance with results from studies using a standard stop-signal task, but in which stop-signal occurrence was nonetheless predictable. For example, striatal activation during successful stopping was observed by Aron & Poldrack [8], who presented a stop-signal once in every four trials, and by Vink et al. [7], [13], who manipulated the number of Go trials between two consecutive Stop trials. Furthermore, activation of the right striatum increased with the probability of having to stop (Fig. 6A), in line with our previous findings [7], [13]. We extended these initial observations by showing that not only the striatum was activated as a function of stop-signal probability, but almost the complete network associated with stopping [8], [41], including the supplementary motor complex (SMC), right inferior frontal cortex (rIFC), anterior cingulate cortex, and bilateral parietal regions. Furthermore, a recent study investigating preparation of inhibition, by comparing activation related to “uncertain” go signals (i.e. those infrequently followed by a stop-signal) with “certain” go signals, also found activation of the SMC and rIFC [42]. These findings suggest that regions commonly associated with outright stopping are in fact also activated during preparation for stopping. In addition, our psychophysiological interaction (PPI) analyses showed that activation of the striatum during successful versus unsuccessful stopping was positively coupled with activation in SMC and rIFC (among other regions) (Fig. 4), providing indirect evidence suggesting that the striatum induces response slowing for improved stopping performance. The reverse PPI, testing whether SMC and rIFC activation during successful stopping was positively coupled with the striatum, was not significant, in line with PPI findings from Duann et al. [43]. Note that the absence of a change in coupling in the reverse PPI is not necessarily odd, because PPIs are not symmetrical (i.e. regression of the interaction between activity in area 1 and context A onto the time series of area 2 is not equal to regression of the interaction between activity in area 2 and context A onto the time series of area 1) [44].

Our results are not consistent with the response build-up hypothesis, proposed by Aron and Poldrack (2006). They argue that striatum activation during successful versus unsuccessful stopping reflects a faster Go response build-up during unsuccessful Stop trials, making it more difficult to inhibit a response. As there is no overt response during successful Stop trials, a direct comparison between successful and unsuccessful Stop trials based on response build-up speed is not possible. Therefore, they tested this hypothesis indirectly by contrasting unsuccessful Stop trials with Go trials with matched response times. They found no striatum activation and interpreted this as showing that response build up is faster during unsuccessful than successful Stop trials. However, in doing so they may also have matched for low stop-signal anticipation (see Fig. 2A). That is, a lack of anticipation probably results in failing to stop. Here, we tested the response build-up hypothesis directly by assessing the parametric effect of Go response time on neural activation. Striatum activation did not increase as a function of response time, but activation of left M1 and left and right superior parietal lobe did (Fig. 6B). This is in line with findings from a study showing that anticipatory response slowing is more likely to be explained by active braking of M1 corticospinal neurons than a slower response build-up [45]. We therefore conclude that striatum activation unlikely reflects an index of response slowing.

Taken together, the present findings indicate that the striatum is a critical node in the neural network associated with stopping planned responses: the data support a role for the striatum in suppression of M1 and anticipation of a stop-signal occurring. Suppression of M1 corticospinal neurons not only occurs after a stop-signal is presented (i.e. *reactive* inhibitory control) [46], [47], but also before presentation of a stop-signal (i.e. *proactive* inhibitory control) [45], [48]. Such a distinction between reactive and proactive mechanisms not only holds for inhibitory control. In fact, a recent influential theory [49], termed the dual mechanisms of control (DMC) account, postulates that cognitive control in general varies along a reactive-proactive continuum. Specifically, proactive control serves an early selection mechanism that can be activated by predictive contextual cues as well as by endogenous signals. It involves anticipation and prevention of interference before it occurs. On the other hand, reactive control can be thought of as a late correction mechanism, triggered by interfering stimuli (e.g. a stop-signal). It relies on stimulus detection and interference resolution. In the following, we will discuss how the striatum may play a role in reactive and proactive inhibitory control.

It is generally agreed that the basal ganglia are important for reactive inhibitory control, but most studies link this function to the subthalamic nucleus rather than the striatum. Foremost, cortical signals conducted via the STN (i.e. via the hyperdirect pathway) reach the basal ganglia output nuclei faster than signals conducted via the striatum (i.e. via the direct and indirect pathways) [50], making the hyperdirect pathway through the STN a stronger candidate for reactive inhibitory control [8]. Other findings implicating the STN in reactive inhibitory control include a relation between shorter stop-signal reaction times (SSRT, a measure of reactive inhibitory control) and stronger STN activation in functional neuroimaging studies [8], [51], longer SSRTs after lesioning the STN in rodents [52], and shorter SSRTs during deep-brain stimulation of the STN in Parkinson's disease patients [53]. Finally, STN activity associated with reactive inhibitory control occurs early enough to influence movements [54]. However, the advantage of signal conduction via the STN over signal conduction via the striatum in terms of time (∼22 ms) is small relative to the SSRT (which is typically 200–250 ms) [6]. Furthermore, striatum activation has also been associated with short SSRTs [12]. Also, as Robbins [55] points out, lesions of the striatum impact SSRT performance more selectively than STN lesions do [21], [52]. Finally, functional neuroimaging studies observe striatum activation, but no STN activation, during successful versus unsuccessful stopping [7], [8]. If inhibition of M1 acts via the striatum, it probably depends upon the indirect pathway that competes with the direct pathway in a push-pull fashion to adjust the amount of inhibitory activity in the basal ganglia output nuclei to brake or facilitate cortically initiated actions, respectively [56], [57], [58]. Although these results implicate the striatum in reactive inhibitory control, the evidence is at best indirect. More direct evidence, showing that activity in the striatum during successful Stop trials modulates before SSRT [3], [4], [59] is lacking.

The role of the striatum in inhibitory control may be much more proactive. Indeed, neurophysiological findings in monkeys implicate striatal neurons in prospective coding of future events, possibly reflecting outcome-oriented behavioral modulation [60], [61], [62]. This is consistent with data from our previous studies, showing that striatum activation was associated with proactive adjustments of response strategies, such as response slowing to improve stopping performance [7], [13]. The striatum exerts its proactive control possibly by modulating the response threshold in M1 [45], [63], [64]. Based on our data, we suggest that this modulation may occur via SMC or rIFC, or both. First, in addition to a coupling between left striatum activation and left M1 deactivation, we found a positive coupling between the striatum and the SMC, and between the striatum and the rIFC. Second, the most significant local maxima in the striatum during stop-signal anticipation and successful stopping (which were used as seeds in the PPI analysis) were located in the anterior putamen. This part of the striatum mediates the cortico-basal ganglia loops through SMC and rIFC. In contrast, we did not find an effect of stop-signal probability in the the posterior part of the putamen that conveys cortico-basal ganglia loops through the primary and premotor cortices [65], [66], [67], [68]. Third, the striatum modulating activity in M1 via SMC or rIFC, rather than SMC or rIFC modulating activity in M1 via the striatum would also be consistent with two recent paired-pulse TMS studies, showing that SMC and rIFC can exert inhibitory control over M1 directly [69], [70]. We speculate that the striatum signals the current context to the cortex to guide behavior, for example, to enhance monitoring of the stop-signal by the rIFC and rTPJ [24] or to induce response time adjustments via the rIFC and SMC, given that stimulation of these areas improves stopping performance by response slowing [25], [26]. Note that the striatum may signal the cortex not only through cortico-basal ganglia loops. Since the striatum harbors the main input to the midbrain dopamine neurons [71], it may also modulate the dopaminergic projections to the cortex. In sum, our data together with the studies discussed above suggest that the striatum is involved in proactive inhibitory control and possibly modulates activity in M1 via SMC and rIFC.

A potential caveat of this study is that PPI analyses are limited in drawing conclusions about the interactions between brain regions in complex neural networks [36], [44]. For instance, it is impossible to determine whether the contribution of one area (e.g. left striatum) onto another (e.g. left M1) is direct, whether the contribution acts via a third structure (e.g. SMC), or whether a third structure (e.g. right orbitofrontal cortex) provides condition-specific input to the two areas (e.g. left striatum and left M1) implicated in the PPI. The present results, therefore, do not allow strong conclusions about the precise pathway via which the striatum contributes to suppression of M1 corticospinal neurons. On the positive side, the results from the present study provide several testable models of how inhibitory control is implemented in the brain. These models can be tested in future studies with more sophisticated effective connectivity analyses, such as dynamic causal modeling and Granger causality analysis.

Another issue that invites further investigation is that our SSRT estimates are longer than usual. Although the SSRT in the standard manual stop-signal paradigm are typically between 200 and 250 ms, there seems to be no strong theoretical reason to expect SSRT to fall within this range. We speculate that our longer SSRT estimates may be characteristic of our particular version of the stop-signal paradigm. The stop-signal anticipation task involves manipulation of stop-signal probability, which varies from trial-to-trial and is made explicit with a visual cue. Stopping in the stop-signal anticipation task may therefore be harder than in the standard stop-signal paradigm. Indeed, there is a tendency for SSRT to increase with task complexity [72] and information load [73]. It is also possible that the stop-signal used in this study (i.e. the bar stopping automatically) was less intense than the stop-signal usually used (e.g. a loud auditory tone), given that previous studies have shown that SSRT increases with a reduction in stop-signal salience [74], [75].

In sum, this study demonstrates that the striatum plays a crucial role in stopping planned responses. We propose that this role entails proactive inhibitory control over response-related activity in M1, most likely achieved in interaction with SMC and rIFC, in order to induce behavioral adjustments that improve stopping performance.

## Supporting Information

Table S1 Local maxima of brain activation for StopSuccess vs StopFailure and StopSuccess vs Go.
(0.28 MB DOC)

Table S2 Local maxima of the psychophysiological interaction analyses.
(0.22 MB DOC)

Table S3 Local maxima of brain activation for the parametric effect of stop-signal probability and response time on Go.
(0.16 MB DOC)
