# Supplementary material for: On the Role of the Striatum in Response Inhibition
Source: PLoS One. 2010 Nov 4;5(11):e13848. doi: 10.1371/journal.pone.0013848 (PMC2973972; doi:10.1371/journal.pone.0013848)
Supplement: Table S1 — Local maxima of brain activation for StopSuccess vs StopFailure and StopSuccess vs Go. (0.28 MB DOC) [file pone.0013848.s001.doc]

| **Supplementary Table 1** |  |  |  |  |  |
| --- | --- | --- | --- | --- | --- |
|  | Cluster size | X | Y | Z | Z statistic |
| Fig. 3A – StopSuccess > StopFailure |  |  |  |  |  |
| R Putamen | 106 | 28 | 8 | -4 | 5.68 |
|  |  | 20 | 4 | 12 | 4.98 |
|  |  | 28 | 0 | 8 | 4.41 |
|  |  | 32 | -16 | 0 | 4.31 |
| L Putamen | 87 | -28 | 0 | 8 | 5.24 |
|  |  | -20 | 8 | -4 | 5.09 |
|  |  | -24 | -20 | 8 | 4.18 |
| R Precuneus | 19 | 20 | -56 | 40 | 4.69 |
| L/R Superior/Middle occipital & cuneus | 57 | -16 | -92 | 8 | 4.57 |
|  |  | 16 | -92 | 20 | 4.52 |
|  |  | 4 | -84 | 8 | 3.65 |
| R Supramarginal gyrus | 21 | 40 | -32 | 40 | 4.34 |
| R Orbitofrontal/Inferior frontal gyrus | 15 | 40 | 40 | -12 | 3.82 |
|  |  | 28 | 48 | -4 | 3.71 |
|  |  |  |  |  |  |
| Fig. 3A – StopSuccess < StopFailure |  |  |  |  |  |
| L Pre/Postcentral gyrus | 80 | -48 | -20 | 44 | 4.95 |
|  |  |  |  |  |  |
| Fig. 3B – StopSuccess > Go 0% |  |  |  |  |  |
| L/R Inferior/Middle/Superior frontal gyrus & Precentral gyrus & Striatum | 1941 | 32 | 24 | 4 | 7.00 |
|  |  | 52 | 8 | 4 | 6.81 |
|  |  | 36 | 16 | -4 | 6.50 |
|  |  | -36 | 16 | 0 | 6.39 |
|  |  | 44 | 0 | 40 | 6.38 |
|  |  | 40 | 4 | 28 | 6.16 |
|  |  | -28 | 24 | 8 | 5.88 |
|  |  | 56 | 8 | 12 | 5.79 |
|  |  | 32 | 40 | 28 | 5.66 |
|  |  | 8 | 12 | 44 | 5.58 |
|  |  | 24 | 8 | -4 | 5.51 |
|  |  | -20 | 8 | 0 | 5.49 |
|  |  | 16 | 4 | 12 | 5.49 |
|  |  | 12 | 4 | 64 | 5.41 |
|  |  | 4 | 12 | 52 | 5.25 |
|  |  | 8 | 4 | 4 | 5.10 |
|  |  | -4 | 4 | 52 | 5.09 |
|  |  | -8 | -8 | -8 | 4.97 |
|  |  | -44 | 0 | 28 | 4.87 |
|  |  | 28 | 48 | 16 | 4.79 |
|  |  | 32 | 60 | -4 | 4.78 |
|  |  | 24 | 52 | -8 | 4.75 |
|  |  | 20 | 0 | -4 | 4.72 |
|  |  | 8 | -12 | -4 | 4.55 |
|  |  | -52 | 4 | 8 | 4.38 |
|  |  | 48 | 28 | 32 | 4.29 |
|  |  | 8 | 32 | 24 | 4.28 |
|  |  | 32 | 36 | 16 | 4.25 |
|  |  | -36 | 8 | 24 | 4.15 |
|  |  | 28 | 60 | 28 | 4.11 |
|  |  | -12 | 12 | 36 | 4.06 |
|  |  | -36 | -8 | 48 | 4.05 |
|  |  | -16 | -4 | 12 | 4.05 |
|  |  | 4 | -24 | -8 | 3.94 |
|  |  | -32 | 0 | 4 | 3.83 |
|  |  | 44 | 44 | -12 | 3.74 |
|  |  | -40 | -8 | 40 | 3.67 |
|  |  | 40 | 48 | 4 | 3.59 |
|  |  | -56 | 4 | 36 | 3.49 |
|  |  | -12 | -12 | 0 | 3.25 |
| R Inferior/Middle/Superior temporal gyrus, Angular gyrus & Middle occipital gyrus | 815 | 56 | -44 | 28 | 6.72 |
|  |  | 60 | -40 | 20 | 5.81 |
|  |  | 64 | -40 | 8 | 5.58 |
|  |  | 36 | -72 | 16 | 5.54 |
|  |  | 28 | -60 | 40 | 5.24 |
|  |  | 44 | -48 | 32 | 5.05 |
|  |  | 40 | -40 | 36 | 4.83 |
|  |  | 48 | -68 | -4 | 4.82 |
|  |  | 28 | -48 | 36 | 4.79 |
|  |  | 56 | -44 | 0 | 4.77 |
|  |  | 44 | -40 | 4 | 4.71 |
|  |  | 64 | -24 | 24 | 4.54 |
|  |  | 40 | -60 | 0 | 3.85 |
|  |  | 8 | -68 | 52 | 3.83 |
|  |  | 12 | -68 | 44 | 3.78 |
| L Supramarginal/Angular/Superior temporal gyrus | 264 | -64 | -40 | 24 | 4.74 |
|  |  | -56 | -44 | 44 | 4.61 |
|  |  | -52 | -48 | 12 | 4.26 |
|  |  | -32 | -44 | 36 | 4.25 |
|  |  | -44 | -40 | 36 | 4.24 |
|  |  | -48 | -40 | 44 | 4.23 |
|  |  | -28 | -72 | 16 | 4.04 |
|  |  | -32 | -76 | 8 | 3.93 |
|  |  | -24 | -64 | 36 | 3.89 |
|  |  | -40 | -56 | 48 | 3.55 |
| L Middle frontal gyrus | 90 | -32 | 40 | 24 | 4.61 |
|  |  | -36 | 48 | 28 | 4.31 |
|  |  | -36 | 44 | 36 | 4.29 |
|  |  | -32 | 60 | 20 | 3.80 |
| R Inferior temporal gyrus | 26 | 48 | -24 | -12 | 4.05 |
|  |  | 60 | -24 | -8 | 3.72 |
|  |  |  |  |  |  |
| Fig. 3B – StopSuccess < Go 0% |  |  |  |  |  |
| L/R Posterior cingulate gyrus, Parahippocampal gyrus, Hippocampus | 1083 | -4 | -64 | 12 | 6.71 |
|  |  | 12 | -56 | 12 | 6.42 |
|  |  | -24 | -36 | -20 | 6.11 |
|  |  | -20 | -52 | 0 | 5.94 |
|  |  | 12 | -48 | -4 | 5.94 |
|  |  | -8 | -56 | 4 | 5.94 |
|  |  | -4 | -52 | 24 | 5.88 |
|  |  | -12 | -52 | -4 | 5.67 |
|  |  | -20 | -12 | -20 | 5.42 |
|  |  | -24 | -20 | -20 | 5.40 |
|  |  | 0 | -68 | 28 | 5.32 |
|  |  | 24 | -20 | -20 | 5.17 |
|  |  | 24 | -52 | 4 | 4.92 |
|  |  | 32 | -48 | -4 | 4.84 |
|  |  | 28 | -56 | 12 | 4.83 |
|  |  | -12 | -40 | -12 | 4.82 |
|  |  | 32 | -28 | -16 | 4.62 |
|  |  | 16 | -36 | -12 | 4.48 |
|  |  | 24 | -40 | 4 | 4.33 |
|  |  | 20 | -40 | -24 | 4.14 |
|  |  | 32 | -4 | -24 | 3.57 |
| L Angular gyrus & Middle occipital gyrus | 136 | -44 | -72 | 24 | 6.23 |
|  |  | -48 | -68 | 16 | 5.88 |
|  |  | -36 | -80 | 36 | 5.71 |
| L/R Superior frontal gyrus | 415 | -4 | 64 | -4 | 6.20 |
|  |  | 4 | 56 | -8 | 5.93 |
|  |  | 0 | 20 | -8 | 4.88 |
|  |  | 0 | 68 | 24 | 4.85 |
|  |  | -4 | 32 | -12 | 4.40 |
|  |  | -8 | 28 | 4 | 4.32 |
|  |  | -12 | 36 | -8 | 4.10 |
|  |  | -16 | 64 | 4 | 4.08 |
|  |  | 0 | 8 | -12 | 3.74 |
|  |  | -4 | 56 | 16 | 3.72 |
|  |  | 4 | 52 | 48 | 3.32 |
| L Pre/Postcentral gyrus | 162 | -52 | -16 | 48 | 5.94 |
|  |  | -36 | -28 | 44 | 5.09 |
|  |  | -32 | -32 | 64 | 4.55 |
|  |  | -16 | -40 | 68 | 4.00 |
| L Middle frontal gyrus | 140 | -20 | 24 | 44 | 5.38 |
|  |  | -4 | 44 | 52 | 3.60 |
|  |  | -24 | 16 | 28 | 3.41 |
| R Middle temporal gyrus | 19 | 44 | 12 | -36 | 4.82 |
|  |  | 56 | 4 | -28 | 3.67 |
| R Angular gyrus & Middle occipital gyrus | 37 | 52 | -72 | 24 | 4.76 |
|  |  | 52 | -64 | 16 | 4.43 |
| L Middle temporal gyrus | 38 | -48 | -4 | -24 | 4.76 |
|  |  | -56 | -8 | -20 | 4.70 |
|  |  | -44 | 12 | -36 | 3.72 |
| R Middle frontal gyrus | 17 | 20 | 36 | 44 | 4.36 |
| L Inferior temporal gyrus | 15 | -60 | -48 | -16 | 4.10 |
|  |  | -52 | -36 | -20 | 3.61 |
| R Postcentral gyrus | 32 | 28 | -32 | 56 | 3.89 |
|  |  | 16 | -36 | 72 | 3.59 |
