# Supplementary material for: On the Role of the Striatum in Response Inhibition
Source: PLoS One. 2010 Nov 4;5(11):e13848. doi: 10.1371/journal.pone.0013848 (PMC2973972; doi:10.1371/journal.pone.0013848)
Supplement: Table S2 — Local maxima of the psychophysiological interaction analyses. (0.22 MB DOC) [file pone.0013848.s002.doc]

| **Supplementary Table 2** |  |  |  |  |  |
| --- | --- | --- | --- | --- | --- |
|  | Cluster size | X | Y | Z | Z statistic |
| Fig. 4A – Positive PPI seeded in -20 8 -8 |  |  |  |  |  |
| R Superior frontal gyrus ^1^ | 172 | 16 | 8 | 64 | 4.74 |
|  |  | 12 | 0 | 68 | 4.32 |
|  |  | 4 | 24 | 40 | 4.30 |
|  |  | 8 | 8 | 48 | 4.00 |
|  |  | -4 | 0 | 48 | 3.71 |
|  |  | 0 | 0 | 60 | 3.65 |
| R Inferior/Middle frontal gyrus/Insular cortex | 235 | 44 | 20 | 4 | 4.52 |
|  |  | 40 | 8 | 24 | 4.48 |
|  |  | 60 | 16 | 4 | 4.43 |
|  |  | 40 | 20 | -8 | 4.34 |
|  |  | 44 | 4 | 32 | 4.06 |
|  |  | 48 | 0 | 44 | 3.96 |
|  |  | 56 | 20 | 24 | 3.85 |
|  |  | 48 | 32 | 28 | 3.67 |
|  |  | 40 | 52 | 24 | 3.56 |
|  |  | 48 | 20 | 16 | 3.54 |
|  |  | 40 | 16 | 36 | 3.49 |
|  |  | 44 | 8 | 12 | 3.40 |
|  |  | 36 | 28 | 24 | 3.39 |
|  |  | 32 | 52 | 16 | 3.35 |
|  |  | 32 | 24 | 16 | 3.34 |
|  |  | 32 | 44 | 28 | 3.31 |
| L Middle frontal gyrus | 22 | -32 | 40 | 28 | 4.51 |
| R Inferior/Middle/Superior temporal gyrus, Angular gyrus & Middle occipital gyrus | 80 | 56 | -48 | -8 | 4.05 |
|  |  | 60 | -48 | 12 | 3.79 |
|  |  | 40 | -68 | -4 | 3.74 |
|  |  | 48 | -44 | 32 | 3.64 |
|  |  | 52 | -64 | -8 | 3.57 |
|  |  | 64 | -52 | 24 | 3.39 |
|  |  | 44 | -56 | -12 | 3.36 |
| R Putamen | 16 | 24 | 8 | -4 | 3.98 |
|  |  | 20 | -8 | -4 | 3.17 |
| R Middle occipital gyrus | 19 | 36 | -72 | 20 | 3.91 |
|  |  |  |  |  |  |
| Fig. 4A – Negative PPI seeded in -20 8 -8 |  |  |  |  |  |
| L Lingual gyrus & Precuneus | 49 | -8 | -60 | 4 | 4.32 |
|  |  | -4 | -72 | 24 | 3.24 |
| R Lingual gyrus & Precuneus | 60 | 12 | -56 | 12 | 4.22 |
|  |  | 24 | -52 | 4 | 3.82 |
|  |  | 16 | -48 | -8 | 3.80 |
|  |  | 8 | -68 | 16 | 3.30 |
| L Pre/Postcentral gyrus | 15 | -20 | -32 | 60 | 4.15 |
| R Insular cortex | 15 | 44 | -16 | 4 | 3.95 |
|  |  | 48 | -12 | 16 | 3.71 |
| L Middle frontal gyrus | 28 | -24 | 20 | 52 | 3.93 |
| L Middle occipital gyrus | 29 | -48 | -76 | 16 | 3.66 |
|  |  |  |  |  |  |
| Fig. 4B – Positive PPI seeded in -28 0 8 |  |  |  |  |  |
| R Superior parietal / Angular gyrus | 16 | 28 | -60 | 40 | 4.62 |
|  |  | 32 | -68 | 48 | 3.36 |
| L Superior parietal gyrus | 24 | -28 | -60 | 48 | 4.28 |
|  |  | -28 | -52 | 36 | 3.89 |
| R Inferior frontal /Orbitofrontal gyrus | 17 | 40 | 48 | 0 | 4.26 |
| R Angular gyrus | 37 | 52 | -48 | 32 | 4.24 |
| R Superior / Middle occipital gyrus | 60 | 24 | -76 | 36 | 4.22 |
|  |  | 28 | -80 | 24 | 4.00 |
|  |  | 36 | -76 | 12 | 3.78 |
|  |  | 48 | -72 | 0 | 3.42 |
| R Inferior frontal gyrus | 32 | 44 | 20 | 0 | 4.03 |
|  |  | 36 | 8 | 20 | 3.84 |
|  |  | 52 | 12 | 12 | 3.34 |
| R Middle frontal gyrus | 17 | 28 | -4 | 56 | 3.70 |
|  |  | 36 | 8 | 48 | 3.24 |
| R Superior frontal gyrus ^1^ | 24 | 0 | 8 | 52 | 3.60 |
|  |  | 4 | 0 | 48 | 3.50 |
|  |  | 8 | 16 | 56 | 3.29 |
|  |  |  |  |  |  |
| Fig. 4C – Positive PPI seeded in 28 8 -4 |  |  |  |  |  |
| R Superior frontal / Cingulate gyrus ^1^ | 98 | 8 | 0 | 68 | 5.27 |
|  |  | 0 | 4 | 48 | 4.79 |
|  |  | 4 | 16 | 44 | 3.74 |
| L Inferior frontal gyrus / Insular cortex | 52 | -44 | 12 | -4 | 5.01 |
|  |  | -28 | 24 | 8 | 3.62 |
|  |  | -60 | 8 | 0 | 3.31 |
| R Middle temporal/Inferior temporal/  Angular/Supramarginal gyrus | 124 | 52 | -44 | 4 | 4.99 |
|  |  | 60 | -44 | 8 | 4.90 |
|  |  | 48 | -52 | -8 | 3.99 |
|  |  | 56 | -48 | 20 | 3.91 |
|  |  | 56 | -44 | -8 | 3.88 |
|  |  | 64 | -40 | 20 | 3.86 |
|  |  | 48 | -60 | -4 | 3.73 |
| R Inferior frontal gyrus/Insular cortex/Putamen | 132 | 44 | 16 | -8 | 4.81 |
|  |  | 28 | 16 | 4 | 3.92 |
|  |  | 36 | 28 | -4 | 3.92 |
|  |  | 28 | 20 | -12 | 3.62 |
|  |  | 20 | 16 | -8 | 3.45 |
|  |  | 48 | 12 | 12 | 3.44 |
| R Middle occipital gyrus | 30 | 32 | -72 | 20 | 4.71 |
|  |  | 24 | -68 | 32 | 3.84 |
|  |  | 28 | -60 | 40 | 3.21 |
| R Inferior frontal gyrus | 22 | 48 | 16 | 24 | 3.77 |
| R Precentral gyrus | 23 | 40 | 0 | 36 | 3.69 |
|  |  |  |  |  |  |
| Fig. 4C – Negative PPI seeded in 28 8 -4 |  |  |  |  |  |
| R Lingual gyrus | 44 | 32 | -48 | 0 | 4.58 |
|  |  | 24 | -40 | 12 | 4.13 |
| L/R Precuneus/Lingual/Cingulate gyrus | 71 | -16 | -52 | 12 | 4.23 |
|  |  | 8 | -64 | 24 | 4.02 |
|  |  | 4 | -64 | 32 | 3.95 |
|  |  | 4 | -56 | 20 | 3.73 |
|  |  | -12 | -60 | 24 | 3.26 |
| L/R Orbitofrontal gyrus | 17 | 0 | 64 | 0 | 4.00 |
|  |  |  |  |  |  |
| Fig. 4D – Positive PPI seeded in 20 4 12 |  |  |  |  |  |
| R Cingulate gyrus | 21 | 8 | 20 | 36 | 4.04 |
|  |  |  |  |  |  |
| Fig. 4D – Negative PPI seeded in 20 4 12 |  |  |  |  |  |
| L Middle occipital gyrus | 15 | -44 | -76 | 16 | 3.85 |
|  |  | -40 | -64 | 16 | 3.40 |
|  |  |  |  |  |  |

^1^ This cluster includes the supplementary motor complex (SMC)
