# Supplementary material for: On the Role of the Striatum in Response Inhibition
Source: PLoS One. 2010 Nov 4;5(11):e13848. doi: 10.1371/journal.pone.0013848 (PMC2973972; doi:10.1371/journal.pone.0013848)
Supplement: Table S3 — Local maxima of brain activation for the parametric effect of stop-signal probability and response time on Go. (0.16 MB DOC) [file pone.0013848.s003.doc]

| **Supplementary Table 3** |  |  |  |  |  |
| --- | --- | --- | --- | --- | --- |
|  | Cluster size | X | Y | Z | Z statistic |
| Fig. 5A – Positive parametric effect of stop-signal probability on Go | | | | | |
| R Inferior frontal/Precentral/Insular gyrus | 164 | 56 | 8 | 24 | 4.78 |
|  |  | 48 | 12 | 4 | 4.67 |
|  |  | 40 | 12 | 28 | 4.30 |
|  |  | 48 | 8 | 32 | 4.15 |
|  |  | 44 | 8 | -4 | 4.12 |
|  |  | 56 | 8 | 36 | 4.01 |
|  |  | 60 | 12 | 8 | 3.47 |
|  |  | 44 | 0 | 24 | 3.41 |
|  |  | 44 | 16 | 40 | 3.17 |
| R Precentral/Superior frontal gyrus | 56 | 20 | -16 | 64 | 4.37 |
|  |  | 24 | -4 | 64 | 4.06 |
|  |  | 32 | -8 | 48 | 3.92 |
|  |  | 20 | 12 | 60 | 3.35 |
|  |  | 12 | 8 | 64 | 3.31 |
| L Superior occipital gyrus | 25 | -28 | -64 | 28 | 4.34 |
| L Supramarginal/Angular/Superior temporal gyrus | 182 | -40 | -44 | 40 | 4.33 |
|  |  | -48 | -32 | 40 | 4.29 |
|  |  | -48 | -40 | 36 | 4.14 |
|  |  | -40 | -48 | 32 | 4.14 |
|  |  | -60 | -32 | 32 | 4.10 |
|  |  | -60 | -28 | 24 | 3.74 |
|  |  | -60 | -48 | 16 | 3.73 |
|  |  | -60 | -20 | 20 | 3.59 |
|  |  | -44 | -60 | 40 | 3.44 |
|  |  | -60 | -48 | 32 | 3.32 |
|  |  | -36 | -60 | 48 | 3.29 |
| R Putamen/Pallidum | 36 | 24 | 16 | -4 | 4.25 |
|  |  | 28 | 0 | 8 | 3.56 |
|  |  | 16 | -4 | -4 | 3.56 |
|  |  | 32 | 4 | 0 | 3.39 |
| L Precentral gyrus | 17 | -56 | 8 | 20 | 4.23 |
|  |  | -44 | 0 | 20 | 3.47 |
| L/R Cingulate/Superior frontal gyrus ^1^ | 106 | 0 | 8 | 44 | 4.23 |
|  |  | 8 | 4 | 40 | 4.18 |
|  |  | 8 | -8 | 40 | 3.82 |
|  |  | -12 | 12 | 36 | 3.76 |
|  |  | 4 | -4 | 56 | 3.63 |
| L Superior frontal gyrus | 40 | -20 | -8 | 68 | 4.21 |
|  |  | -16 | 4 | 64 | 3.92 |
|  |  | -28 | -4 | 64 | 3.58 |
| R Supramarginal/Angular/Superior temporal gyrus | 82 | 56 | -40 | 28 | 4.09 |
|  |  | 64 | -36 | 20 | 4.00 |
|  |  | 64 | -32 | 36 | 3.93 |
|  |  | 56 | -36 | 48 | 3.87 |
|  |  | 44 | -28 | 36 | 3.80 |
|  |  | 52 | -44 | 52 | 3.58 |
| R Insular cortex / Inferior frontal gyrus | 18 | 32 | 32 | 8 | 4.08 |
|  |  | 44 | 32 | 20 | 3.31 |
| L Middle frontal gyrus | 32 | -40 | 40 | 32 | 3.84 |
|  |  | -36 | 48 | 16 | 3.79 |
|  |  | -44 | 36 | 24 | 3.75 |
|  |  | -28 | 52 | 28 | 3.43 |
| R Superior parietal / Angular gyrus | 63 | 32 | -56 | 48 | 3.76 |
|  |  | 24 | -64 | 44 | 3.57 |
|  |  | 24 | -68 | 32 | 3.42 |
| L Precuneus / Superior parietal gyrus | 23 | -8 | -68 | 44 | 3.64 |
|  |  | -8 | -80 | 44 | 3.47 |
|  |  |  |  |  |  |
| Fig. 5A – Negative parametric effect of stop-signal probability on Go | | | | | |
| L/R Superior frontal gyrus | 25 | -4 | 60 | -8 | 3.95 |
|  |  | 4 | 52 | -16 | 3.57 |
|  |  |  |  |  |  |
| Fig. 5B – Positive parametric effect of response time on Go | | | | | |
| R Superior parietal gyrus | 68 | 28 | -56 | 60 | 4.49 |
|  |  | 12 | -68 | 60 | 4.12 |
|  |  | 12 | -60 | 64 | 4.11 |
|  |  | 24 | -68 | 52 | 4.02 |
| L Pre/Post-central gyrus | 56 | -16 | -12 | 52 | 4.45 |
|  |  | -8 | -12 | 56 | 4.22 |
|  |  | -28 | -16 | 56 | 3.96 |
|  |  | -16 | -20 | 56 | 3.87 |
|  |  | -28 | -12 | 44 | 3.68 |
| L Superior parietal gyrus | 64 | -24 | -56 | 56 | 4.26 |
|  |  | -12 | -60 | 48 | 4.21 |
|  |  |  |  |  |  |
| Fig. 5B – Negative parametric effect of response time on Go | | | | | |
| L/R Anterior cingulate gyrus | 126 | -4 | 36 | 12 | 5.39 |
|  |  | -4 | 28 | 20 | 4.83 |
|  |  | -4 | 32 | -12 | 3.72 |
| R Insular cortex | 20 | 36 | 16 | -16 | 4.47 |
|  |  |  |  |  |  |

^1^ This cluster includes the supplementary motor complex (SMC)
